# Ultrasonication Improves Solid Phase Synthesis of Peptides Specific for Fibroblast Growth Factor Receptor and for the Protein-Protein Interface RANK-TRAF6

**DOI:** 10.3390/molecules26237349

**Published:** 2021-12-03

**Authors:** Rúben D. M. Silva, João Franco Machado, Kyle Gonçalves, Francisco M. Lucas, Salete Batista, Rita Melo, Tânia S. Morais, João D. G. Correia

**Affiliations:** 1 Centro de Ciências e Tecnologias Nucleares, Instituto Superior Técnico, Universidade de Lisboa, CTN, Estrada Nacional 10 (km 139.7), Bobadela, 2695-066 Loures, Portugal; ruben.silva@ctn.tecnico.ulisboa.pt (R.D.M.S.); joaomachado@campus.ul.pt (J.F.M.); fc48261@alunos.fc.ul.pt (K.G.); franciscolucas99@gmail.com (F.M.L.); salete.baptista@ctn.tecnico.ulisboa.pt (S.B.); ritamelo@ctn.tecnico.ulisboa.pt (R.M.); 2 Centro de Química Estrutural and Departamento de Química e Bioquímica, Faculdade de Ciências, Universidade de Lisboa, Campo Grande, 1749-016 Lisboa, Portugal; 3 Departamento de Engenharia e Ciências Nucleares, Instituto Superior Técnico, Universidade de Lisboa, CTN, Estrada Nacional 10 (km 139.7), Bobadela, 2695-066 Loures, Portugal

**Keywords:** difficult sequences, peptides, solid phase synthesis, sonochemistry and ultrasound

## Abstract

Considering our interest in the use of peptides as potential target-specific drugs or as delivery vectors of metallodrugs for various biomedical applications, it is crucial to explore improved synthetic methodologies to accomplish the highest peptide crude purity in the shortest time possible. Therefore, we compared “classical” fluorenylmethoxycarbonyl (Fmoc)-solid phase peptide synthesis (SPPS) with ultrasound(US)-assisted SPPS based on the preparation of three peptides, namely the fibroblast growth factor receptor 3(FGFR3)-specific peptide Pep1 (VSPPLTLGQLLS-NH_2_) and the novel peptides **Pep2** (RQMATADEA-NH_2_) and **Pep3** (AAVALLPAVLLALLAPRQMATADEA-NH_2_), which are being developed aimed at interfering with the intracellular protein-protein interaction(PPI) RANK-TRAF6. Our results demonstrated that US-assisted SPPS led to a 14-fold (**Pep1**) and 4-fold time reduction (**Pep2**) in peptide assembly compared to the “classical” method. Interestingly, US-assisted SPPS yielded Pep1 in higher purity (82%) than the “classical” SPPS (73%). The significant time reduction combined with high crude peptide purity attained prompted use to apply US-assisted SPPS to the large peptide **Pep3**, which displays a high number of hydrophobic amino acids and homooligo-sequences. Remarkably, the synthesis of this 25-mer peptide was attained during a “working day” (347 min) in moderate purity (approx. 49%). In conclusion, we have reinforced the importance of using US-SPPS towards facilitating the production of peptides in shorter time with increased efficacy in moderate to high crude purity. This is of special importance for long peptides such as the case of **Pep3**.

## 1. Introduction

Owing to our interest in the study of target-specific molecules that can interfere with relevant protein-protein interactions (PPI) or that can act as delivery vectors of metallodrugs for biomedical applications, namely cancer theranostics, we have been actively involved in the design and biological evaluation of relevant targeting peptides [1,2,3,4,5,6]. The latter comprise blood brain barrier-penetrating peptide shuttles, melanocortin-1 receptor (MC1R)-targeting peptides, integrin receptor α_v_β_3_-specific cyclic pentapeptides or fibroblast growth factor receptor (FGFR)-specific peptides. The FGFR, which comprises four identified subtypes (FGFR1-FGFR4), is a transmembrane tyrosine kinase that plays an important role in various signaling pathways, such as those controlling organogenesis as well as those related to tissue reparation and metabolic function [7]. Alterations of FGFR structure, function or expression are often associated with the development and/or progression of many metabolic disorders and cancer [7,8]. Therefore, targeting FGFR has been considered a promising approach for the therapy of various cancers, with many drug candidates reaching clinical trials, namely highly specific molecules such as peptides [8]. Amongst the latter, the peptide VSPPLTLGQLLS-NH_2_ (**Pep1**) binds with high affinity and specificity to the extracellular domain of FGFR3, and was explored for the treatment of human thanatophoric dysplasia and for regulation of lymphangiogenesis [9,10,11].

More recently, some of us explored the possibility of using **Pep1** for the selective delivery of a cytotoxic ruthenium complex, [RuCp(PPh_3_)(2,2′-bipy)][CF_3_SO_3_] (Cp = η^5^-cyclopentadienyl, **TM34**) [12,13,14,15], to highly metastatic breast cancer cells that overexpress the FGFR [2,16]. The ruthenium peptide conjugate **TM34-Pep1** obtained after conjugation was more active against the FGFR-overexpressing breast cancer cells (SKBR3, FGFR+) than against those that did not overexpress that receptor (MDAMB231, FGFR–). These findings highlighted the importance of using FGFR-targeting peptides for selective drug delivery and prompted us to develop further improved cytotoxic conjugates with higher selectivity.

A brief analysis of the amino acid sequence of **Pep1** permits to identify the presence of the hydrophobic amino acids Gly, Leu and Val and of the homooligo-sequence LeuLeu, which may promote aggregation, and empirically include it into the category of the so-called “difficult peptides” [17].

The pioneering work of Takahashi and Shimonishi in the use of ultrasonic waves in Solid Phase Peptide Synthesis (SPPS) and the unprecedented systematic study recently undertaken by Merlino et al. have demonstrated that Ultrasound (US)-assisted SPPS can be placed among the current highly efficient peptide synthetic methodologies [18,19]. Indeed, the authors demonstrated that 9-fluorenylmethyloxycarbonyl (Fmoc)-deprotection yields higher than 95% were achievable with reaction times as low as 2 min by US-assisted SPPS [19]. This resulted in a 92% time reduction as compared to the “classical” method of Fmoc-removal that typically requires at least 25 min. Additionally, the same authors reported considerable time reduction in amide bond formation. Similar relevant results on the application of ultrasonication in peptide synthesis have been reported by other authors [20,21]. Remarkably, sonication does not cause the racemization of sensible residues as clearly demonstrated by Merlino et al. and Wołczański et al. and can be applied to assemble peptides bearing amino acid residues prone to racemization [19,20].

Inspired by these achievements, we decided to prepare **Pep1** using this approach and compare it with the “classical” standard synthetic methodology in the absence of ultrasound irradiation. The success of US-assisted SPPS of **Pep1** was extended to **Pep2** (RQMATADEA-NH_2_) and **Pep3** (AAVALLPAVLLALLAPRQMATADEA-NH_2_), which are being developed aimed at interfering with the intracellular PPI RANK-TRAF6. The latter is recognized as a potential therapeutic target for bone-related diseases, including bone cancer metastasis [22,23,24].

## 2. Results and Discussion

Considering the relevance of **Pep1** as a FGFR inhibitor potentially useful for the treatment of metabolic diseases such as thanatophoric dysplasia or targeted delivery of cytotoxic agents, it is important to further deepen the understanding of these applications and expand to other areas. Therefore, the development of more efficient, clean, and fast synthetic procedures is of outmost importance. In our first attempt to synthesize **Pep1**, we adopted a Fmoc-based SPPS method using a Rink Amide MBHA resin and standard reagents (O-(Benzotriazol-1-yl)-*N*,*N*,*N′*,*N′*-tetramethyluronium hexafluorophosphate, HBTU and *N*,*N*-Diisopropylethylamine, DIPEA) in a microwave (MW)–assisted automated peptide synthesizer. However, this approach revealed unsuccessful, as we could not find the optimal conditions, even after several attempts using the same coupling agent, in which different reagents stoichiometry, coupling times, number of coupling steps and/or temperatures were assayed. Nevertheless, the use of other coupling agents (e.g., HOBT/DIC) might have given a different result.

**Figure 1:**
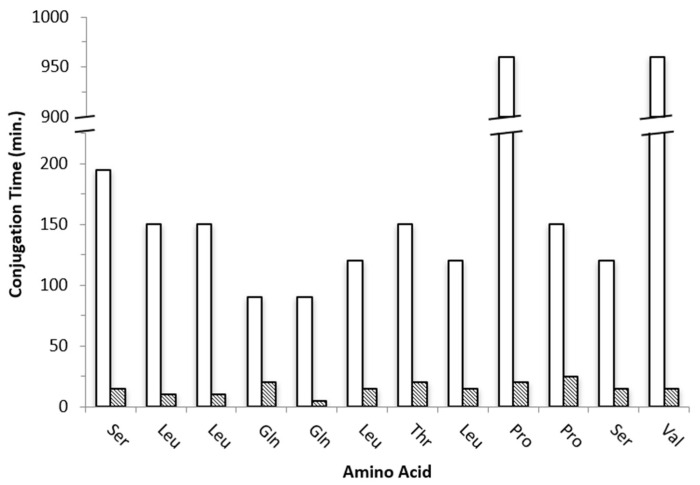
Conjugation times (min) for each amino acid of peptide **Pep1** prepared by “classical” SPPS (white, 3255 min total) and by US-SPPS (dashed, 185 min total).

To overcome this limitation, we then proceeded to the “classical” manual synthetic approach (0.1 mmol scale) in the absence of MW irradiation at room temperature. Unlike the automated synthesis tried above, in the case of the “classical” method adopted, it has been possible to monitor the coupling of each amino acid by performing a step-by-step colorimetric Kaiser test at each deprotection and conjugation reaction [25]. **Pep1** was assembled on the resin using standard optimized Fmoc-deprotection cycles (20% piperidine in DMF, 20 min) and amino acid conjugation cycles with times ranging from 90 min to 960 min at room temperature (Figure 1).

**Figure 2:**
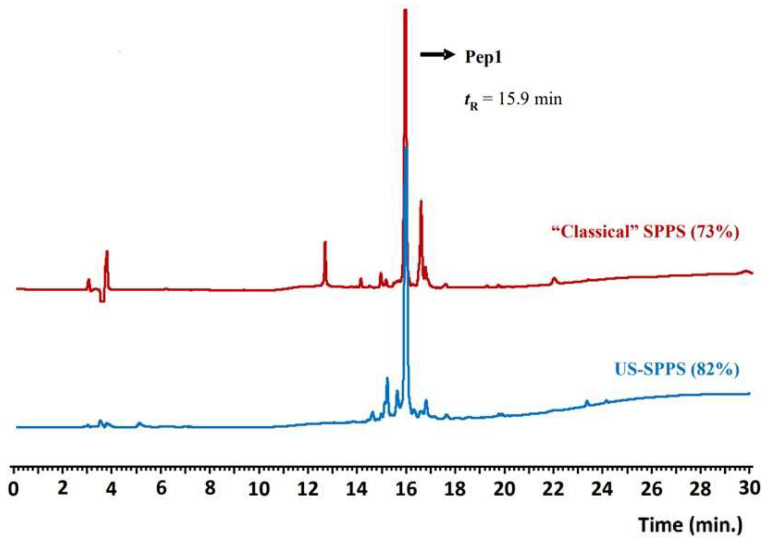
Analytical RP-HPLC chromatograms obtained for crude **Pep1** (***t*_R_** = 15.9 min); comparison between the products obtained after synthesis by “classical” SPPS (red) or US-SPPS (blue). Gradient A (see experimental section).

Cleavage of **Pep1** from the resin, full deprotection, and isolation were achieved with a standard cleavage cocktail (95% TFA/2.5% TIS/2.5% H_2_O) and treatment with ice-cold diethyl ether. The peptide precipitate was recovered by centrifugation, dried under N_2_ stream and dissolved in water. **Pep1** was controlled by analytical reversed-phase (RP) HPLC and characterized by ESI-MS spectrometry, revealing a crude purity of ca. 73% (Table 1, Figure 2). The final yield for the crude peptide was ca. 42% (Table 2).

Although “classical” SPPS gave **Pep1** in moderate crude purity, this approach revealed to be highly demanding and time-consuming as more than 58 h were needed for total peptide assembly on the resin. Aimed at finding a faster route and speed up both the Fmoc-deprotection and amino acid conjugation steps, we explored the combination of SPPS with ultrasonication using a common laboratory ultrasonic water bath. The same experimental conditions described above for the “classical” SPPS method were followed but with controlled temperature (30 ± 5 °C) to avoid overheating due to ultrasound irradiation. With this methodology, the Fmoc-deprotection step could be reduced from 20 to 5 min each (4-fold decrease), and the amino acids conjugation step was attained in only 5 to 25 min per step (Figure 1). Remarkably, **Pep1** was assembled successfully on the resin in approximately 4 h, which means that it can be prepared 14-fold faster than by “classical” SPPS. Moreover, the US-SPPS approach allowed a more efficient and clean synthesis, as the crude product purity increased from 73% to 82%, with less formation of side-products as determined by analytical RP-HPLC and ESI-MS (Figure 2, Table 1). This improvement is highly relevant as it reduces the production costs and facilitates the purification of final peptide **Pep1**. Moreover, the final yield for the crude peptide obtained by US-SPPS (54%) is significantly higher than that observed in “classical” SPPS (42%) (Table 2).

**Figure 3:**
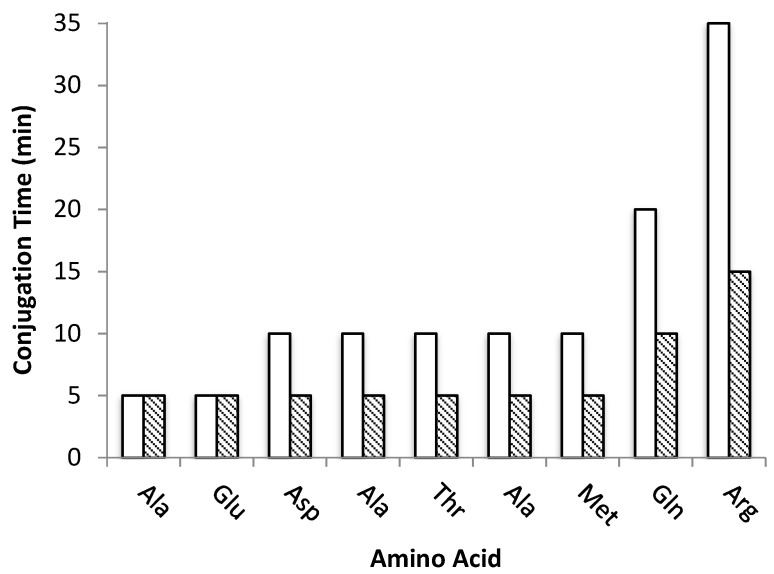
Conjugation times (min) for each amino acid of peptide **Pep2** prepared by “classical” SPPS (white, 115 min total) and US-SPPS (dashed, 65 min total).

This successful accomplishment prompted us to apply the same methodology to the synthesis of the 9-mer peptide RQMATADEA-NH_2_ (**Pep2**), a potential inhibitor of the intracellular PPI RANK-TRAF6 and compare the US-assisted method to the “classical” SPPS under optimized reaction conditions. In the latter strategy, the Fmoc-deprotection step took 25 min per amino acid and conjugation times varied between 5 to 35 min, depending on the amino acid and relative position within the chain (Figure 3). Therefore, the total time for peptide assembly was 365 min.

Interestingly, in the case of US-assisted peptide synthesis under the same conditions, the Fmoc-deprotection step was reduced to 2 min whereas the conjugation step required between 5 to 15 min, which accounts for a total time of 85 min to complete the sequence of **Pep2** (Figure 3). As the peptide chain grows, in the “classical” synthesis an increase on time required to a complete conjugation is observed, while US-assisted required a steady 5 min time per amino acid until the 7th residue (Met).

Similar to **Pep1**, both the Fmoc-deprotection and the conjugation steps in **Pep2** were monitored by the Kaiser test colorimetric assay. Brought together the results showed that there was a 4-fold time reduction to assemble **Pep2** on the resin when the “classical” SPPS method (365 min) was replaced by the US-SPPS (85 min).

The resin-bound peptides, from both the “classical” and US-assisted SPPS synthesis, were cleaved from the resin and isolated as described above and analyzed by RP-HPLC and ESI-MS (Table 1).

**Figure 4:**
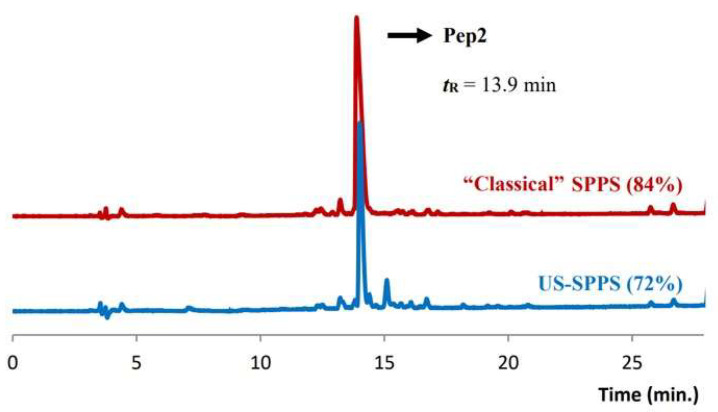
Analytical RP-HPLC chromatograms obtained for crude **Pep2** (***t*_R_** = 13.9 min); comparison between the products obtained by US-SPPS (blue) and “classical” SPPS (red). Gradient B (see experimental section).

Analysis of the chromatographic profiles revealed 84% and 72% purity for the crude peptides obtained by “classical” and US-SPPS, respectively (Figure 4). The lower purity of the peptide obtained by US-SPPS can be tentatively assigned to local overheating phenomena associated to ultrasound irradiation. Similar to what was observed in the synthesis of **Pep1**, the final yield for crude **Pep2** prepared by US-SPPS (49%) was higher than that obtained for the same peptide prepared by “classical” SPPS (32%) (Table 2).

Encouraged by the significant time reduction provided by US-assisted SPPS of **Pep1** and **Pep2**, the same synthetic methodology was applied to the long peptide **Pep3** (A^1^AVALLPAVLLALLAP^16^RQMATADEA-NH_2_), which is a **Pep2** derivative. **Pep3** contains the RANK-TRAF6 potential interfering sequence, **Pep2**, and a long sequence (from Ala^1^ to Pro^16^) with an high number of hydrophobic amino acids (Ala, and Leu) and homooligo-sequences LeuLeu and AlaAla [17]. This latter sequence has been described as a potent cell penetrating peptide by Ye el al. [22]

**Figure 5:**
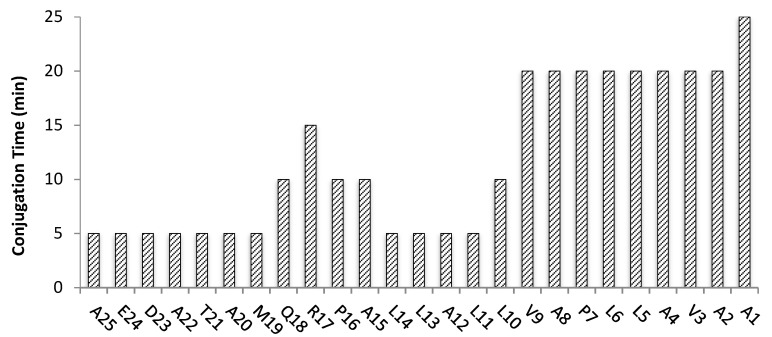
Conjugation times (min) for each amino acid of the 25-mer peptide **Pep3** prepared by US-assisted SPPS. Total conjugation time was 295 min.

Following the optimized reaction conditions used for **Pep2**, the monitored Fmoc-deprotection step took the same time (2 min), whereas the average conjugation time for the first 16 amino acids (from A^25^ to L^10^) and for the last nine amino acid residues (from Val^9^ to Ala^1^) was approximately 7 min and 21 min, respectively (Figure 5). Complete **Pep3** assembly by US-assisted SPPS took 347 min.

Although we did not try the synthesis of the long peptide **Pep3** by “classical” SPPS, it is predictable that it would take much longer based on our experience with the shorter peptides **Pep1** and **Pep2**, where up to 14-fold time reduction required to assemble the complete peptide was observed when ultrasonication was applied.

**Figure 6:**
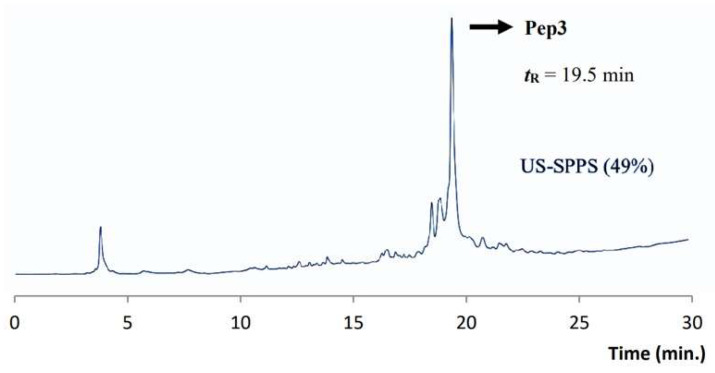
Analytical RP-HPLC chromatogram obtained for crude **Pep3** (***t*_R_** = 19.5 min). Gradient C (see experimental section).

After cleavage and precipitation of crude peptide by standard methods, **Pep3** was characterized by analytical RP-HPLC and ESI-MS (Table 1). **Pep3** was obtained in approx. 49% crude purity as shown by the RP-HPLC trace (Figure 6). The final yield for crude peptide was ca. 19% (Table 2).

## 3. Conclusions

We have compared “classical” SPPS with US-assisted SPPS in the preparation of **Pep1** (VSPPLTLGQLLS-NH_2_) and **Pep2** (RQMATADEA-NH_2_). Remarkably, US-assisted SPPS led to a 14-fold (**Pep1**) and 4-fold time reduction (**Pep2**) in peptide assembly. Interestingly, in the case of **Pep 1**, US-assisted SPPS yielded a crude peptide with higher purity (82%) than that obtained by “classical” SPPS (73%). On the contrary, the purity of **Pep2** prepared by US-SPPS was lower (72%) when compared to “classical” SPPS (84%). This has been tentatively assigned to aspartimide formation during Asp conjugation, which can be most likely circumvented by lowering reaction temperature or using a bulkier side-chain-protecting group for Asp (e.g., 5-N-butyl-5-nonyl, OBno). The significant time reduction combined with high peptide purities (>70%) prompted us to apply US-assisted SPPS to a large peptide (**Pep3**, AAVALLPAVLLALLAPRQMATADEA-NH_2_) with high numbers of hydrophobic amino acids and homooligo-sequences. The synthesis of this 25-mer peptide was accomplished within a “working day” (347 min) in moderate purity (approx. 49%).

In conclusion, we have reinforced the importance of using US-SPPS towards facilitating the production of peptides in shorter time with increased efficacy and moderate to high crude purity. This is of special importance for long peptides containing potential “difficult sequences” such as the case of **Pep3**.

## 4. Materials and Methods

### 4.1. Materials

Dichloromethane (DCM) and *N*,*N*-Dimethylformamide (DMF) were purchased from Honeywell Riedel-de Haen™ and were used without further purification. Trifluoroacetic acid (TFA), triisopropylsilane (TIS), 1,2-ethanedithiol (EDT), piperidine and *N*,*N*-diisopropylethylamine (DIPEA) were purchased from Merck^®^. All Fmoc-L-AA-OH (with the respective side chain protecting group orthogonal to Fmoc-based SPPS) and 2-(1H-Benzotriazol-1-yl)-1,1,3,3-tetramethyluronium hexafluorophosphate (HBTU) were acquired from Novabiochem™ and Iris Biotech GmbH, respectively.

### 4.2. Reactor Vessels for “Classical” and US-Assisted SPPS

“Classical” SPPS (**Pep1** and **Pep2**) was performed in a glass reactor with a porous fritted glass while Ultrasound assisted SPPS (**Pep1**–**Pep3**) was performed using a polymeric reactor (syringe) with an incorporated frit (PP—Reactors 5 mL with PE frit, Multisyntech GmbH) and removable cap to avoid contamination with water from the ultrasound bath. Stirring of the solutions was achieved by N_2_ flow in the case of classical synthesis and ultrasonic irradiation for the US-assisted synthesis. Sonication for SPPS was performed in a Fisherbrand™ S-Series FB15051, (240 × 137 × 150 mm bath dimension and 2.75 L max. volume) ultrasonic water bath by Fisher Scientific. Ultrasonic frequency was 37 kHz. During all procedures the operation water volume was kept at approximately 1.5 L and the temperature kept at 30 ± 5 °C. The ultrasonic output was 200 W, with the peak at 320 W. To identify the zone where cavitation phenomenon was stronger, aluminum foil test was performed. We observed that cavitation was stronger closer to the center of the bath. Therefore, all reactions were performed with the reactor in the middle of the bath and at the same depth [19]. Electrospray ionization mass spectrometry (ESI–MS) was performed on a QITMS instrument (Bruker HCT, Bruker, Billerica, MA, USA) in positive and negative ionization modes, using acetonitrile/water mixture as solvent.

### 4.3. Synthesis of the Peptides

The peptides were obtained by standard Fmoc-based SPPS strategy with Fmoc-protecting group on the α-*N* of the amino acids and orthogonal side chain protecting groups. The following groups were used: *tert*-butyl (tBu) for aspartic acid, serine and tyrosine; tert-butyloxycarbonyl (OtBu) for glutamic acid; pentamethyl-2,3-dihydrobenzofuran-5-sulfonyl (Pbf) for arginine; and trityl (Trt) for glutamine. Peptides were prepared as *C*-terminal amides on a Rink Amide MBHA resin (100–200 mesh). Before conjugation of the first amino acid, the resin was swollen by suspension in excess DMF and gently stirred (ca. 30 min) using an N_2_ flow in the case of classical synthesis or an orbital shaker in the case of US-assisted synthesis.

The syntheses were carried out at 0.1 mmol scale on Rink Amide MBHA resins (100–200 mesh), resin loading: **Pep1** 0.78 mmol/g, **Pep2** and **Pep3** 0.38 mmol/g at room temperature (“classical” SPPS) or at 30 ± 5 °C (US-SPPS).

Fmoc-deprotection was completed after treatment of the resin with a 20% (*v*/*v*) Piperidine/DMF solution (“classical” SPPS: **Pep1** 20 min, **Pep 2** 25 min; US-Assisted SPPS: **Pep1** 5 min; **Pep2** and **Pep3** 2 min). In both synthetic approaches, peptide assembly was accomplished by stepwise addition of Fmoc-L-AA-OH (**Pep1** 5.0 eq, **Pep2** and **Pep 3** 3.5 eq) and HBTU (**Pep1** 5.0 eq, **Pep2** and **Pep 3** 3.5 eq) in DMF solutions in the presence of DIPEA (10 eq) to the resin, during variable periods as presented in Figure 1, Figure 3 and Figure 5. The efficiency of each coupling and deprotection steps were monitored by the Kaiser test [25]. After peptide assembly and final Fmoc-deprotection, cleavage from resin and removal of side chain protecting groups was attained using a standard cleavage cocktail (**Pep1** TFA 95%/TIS 2.5%/water 2.5%, *v*/*v*; **Pep2** and **Pep3** TFA 92.5%/TIS 2.5%/EDT 2.5%/water 2.5%, *v*/*v*). The peptides were precipitated by cold diethyl ether and isolated by centrifugation. The crude peptides (**Pep1–3**) were obtained as white residues, dried under a gentle stream of N_2_ and stored at −20 °C. The crude peptides were characterized by reversed phase high performance liquid chromatography (RP-HPLC) and electrospray ionization mass spectrometry (ESI-MS) (see Supplementary Material).

### 4.4. Kaiser Test Colorimetric Assay Procedure

Kaiser test is a colorimetric assay which identifies the presence of free primary amines in a mixture [25]. In SPPS procedures, this assay is used to qualitatively access the Fmoc-removal/amino acid coupling reaction yields. In brief, a small fraction of the resin beads (5 to 10) is separated from the bulk. To this fraction, 5 μL of 1 mM KCN in H_2_O: pyridine (1:49), 5 μL of 0.3 M ninhydrin in ethanol and 5 μL of 42.5 M phenol in ethanol are added. The mixture is heated at 110 °C for 5 min and the color of the solution/beads is observed. Change in the color of the solution/beads to blue indicates the presence of free primary amines.

### 4.5. RP-HPLC Analysis

Control analytical RP-HPLC analyses were performed on a PerkinElmer LC pump 200 coupled to a PerkinElmer Series 200 UV/Vis Detector. Analytical control of the synthetized peptide samples was achieved on a Supelco Discovery^®^ Bio Wide Pore C18-5 column (250 mm × 4.6 mm, 5 μm) with a flow rate of 1.0 mL min^−1^ and UV detection at λ = 220 nm.

### 4.6. Applied Binary HPLC Gradients

*Eluents*: mobile phase A—0.1% TFA aq.; mobile phase B—0.1%TFA in MeCN.

*Gradient A* (**Pep1**): 0→3 min, 10% B; 3→18 min, 10–100% B; 18→21 min, 100%B; 21→24 min, 100–10% B; 24→25 min, 10% B.

*Gradient B* (**Pep2**): 0→5 min, 10% B; 5→25 min, 10–25% B; 25→27 min, 25–100% B; 27→28 min, 100% B.

*Gradient C* (**Pep3**): 0→5 min, 10% B; 5→25 min, 10–100% B; 25→28 min, 100% B; 28→30 min, 100–10% B.

## Figures and Tables

**Table 1:** Analytical characterization and complete synthesis times for **Pep1**, **Pep2** and **Pep3**.

Peptide/Sequence	Calc. Exact Mass (Da)	Found [ion]	*t*_R_ (Min)/Purity	Total Synthesis (Min)
**Pep1** VSPPLTLGQLLS-NH_2_	C_56_H_98_N_14_O_16_ 1222.7	1223.8 [M+H]^+^ 612.5 [M+2H]^2+^	15.9 *^a^* Cl. 73% US 82%	Cl. 3515 US 250
**Pep2** RQMATADEA-NH_2_	C_38_H_64_N_14_O_15_S 991.09	991.6 [M+H]^+^ 496.3 [M+2H]^2+^	13.9 *^b^* Cl. 84% US 72%	Cl. 365 US 85
**Pep3** AAVALLPAVLLALLAPRQMATADEA-NH2	C_112_H_192_N_30_O_31_S 2489.02	1245.5 [M+2H]^2+^ 830.9 [M+3H]^3+^	19.5 *^c^* US 50%	US 347

*^a^* Gradient A; *^b^* Gradient B; *^c^* Gradient C (see experimental section for gradients). Cl. = Classical SPPS; US = US-assisted SPPS.

**Table 2:** Final yield (%) for crude **Pep1**–**Pep3** obtained by “classical” vs. US-SPPS.

Peptide	Classical SPPS	US-SPPS
**Pep1**	42	54
**Pep2**	32	49
**Pep3**	-	19

## Data Availability

Not applicable.

## Supplementary Materials

The following are available online, Figure S1: Structures of Pep1-Pep3, Figure S2: ESI-MS spectra (positive mode) of Pep1-Pep3 in acetonitrile, with the ionic species found highlighted in red.
