# Supplementary material for: Ultrasonication Improves Solid Phase Synthesis of Peptides Specific for Fibroblast Growth Factor Receptor and for the Protein-Protein Interface RANK-TRAF6
Source: Molecules. 2021 Dec 3;26(23):7349. doi: 10.3390/molecules26237349 (PMC8659051; doi:10.3390/molecules26237349)
Supplement: Supplementary file 1 [file molecules-26-07349-s001.zip › molecules-1453178-supplementary.pdf]

Supplementary Material

Ultrasonication Improves Solid Phase Synthesis of Peptides Specific for Fibroblast Growth Factor Receptor and for the protein-protein interface RANK-TRAF6

Rúben D. M. Silva[§,1], João Franco Machado[§,1,2], Kyle Gonçalves[1], Francisco M. Lucas[1], Salete Batista[1], Rita Melo[1], Tânia S. Morais[2*] and João D. G. Correia[1,3*]

[1] Centro de Ciências e Tecnologias Nucleares, Instituto Superior Técnico, Universidade de Lisboa, CTN, Estrada Nacional 10 (km 139,7), 2695-066 Bobadela LRS, Portugal

[2] Centro de Química Estrutural and Departamento de Química e Bioquímica, Faculdade de Ciências, Universidade de Lisboa, Campo Grande, 1749-016 Lisboa, Portugal.

[3] Departamento de Engenharia e Ciências Nucleares, Instituto Superior Técnico, Universidade de Lisboa, CTN, Estrada Nacional 10 (km 139,7), 2695-066 Bobadela LRS, Portugal

* Correspondence: to: João D. G. Correia (jgalamba@ctn.tecnico.ulisboa.pt) and Tânia S. Morais tsmorais@fc.ul.pt).

§ These authors contributed equally to the work.

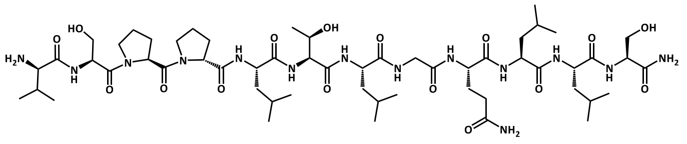

Pep1

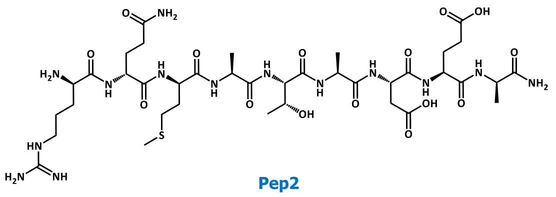

Pep2

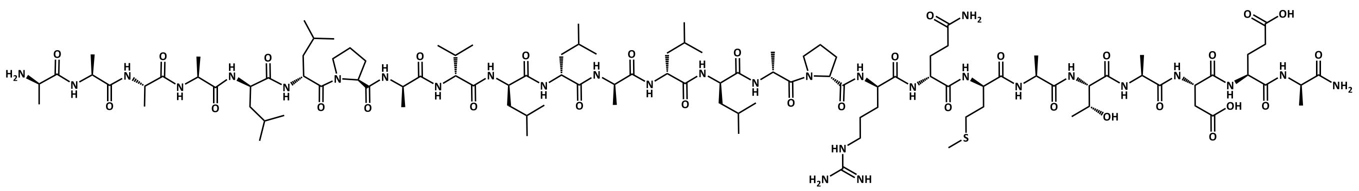

Pep3

Figure S1. Structures of **Pep1**-**Pep3**.

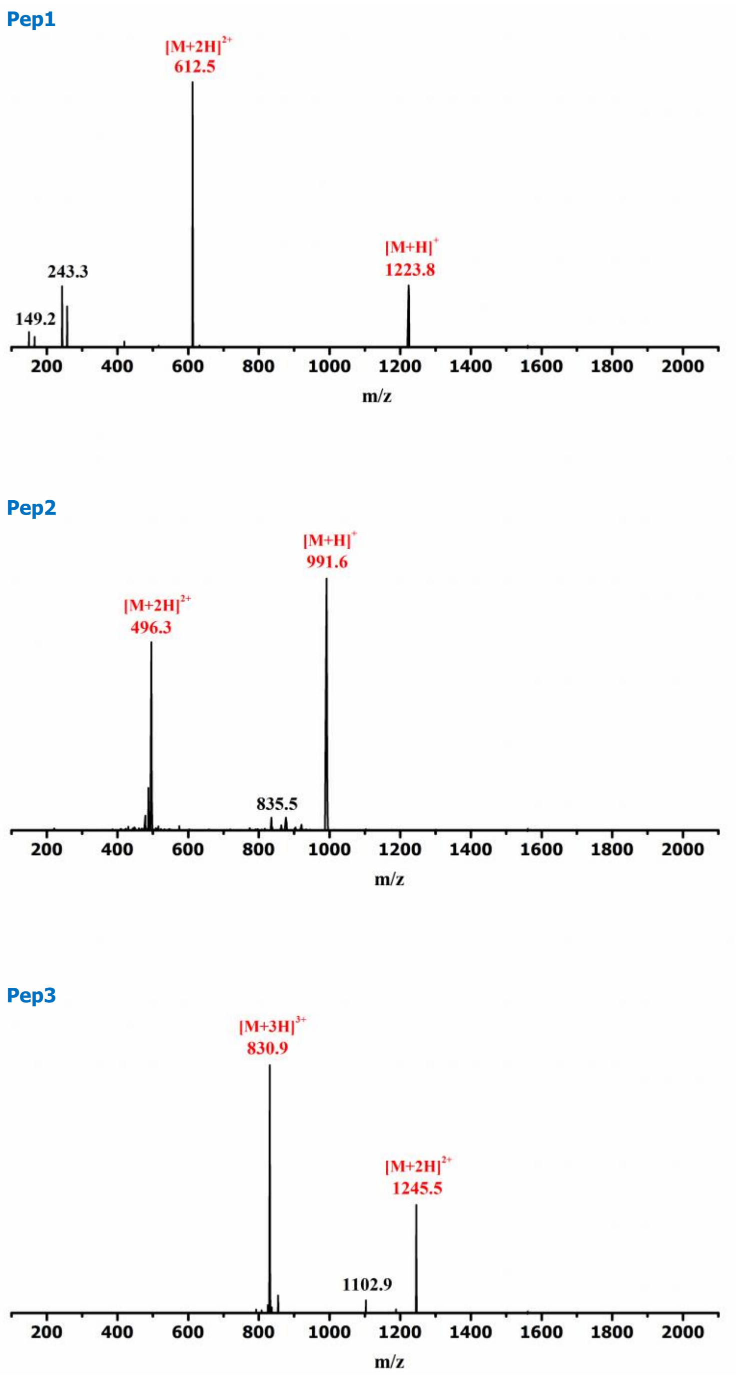

Figure S2. ESI-MS spectra (positive mode) of **Pep1**-**Pep3** in acetonitrile, with the ionic species found highlighted in red.
